# Sulphur‐Acquisition Pathways for Cysteine Synthesis Confer a Fitness Advantage to Bacteria in Plant Extracts

**DOI:** 10.1111/1462-2920.70126

**Published:** 2025-06-17

**Authors:** Kazuya Ishikawa, Saki Yamaguchi, Taketo Tsukaoka, Makoto Tsunoda, Kazuyuki Furuta, Chikara Kaito

**Affiliations:** ^1^ Graduate School of Medicine, Dentistry and Pharmaceutical Sciences Okayama University Okayama Japan; ^2^ Graduate School of Pharmaceutical Sciences The University of Tokyo Bunkyo Tokyo Japan

**Keywords:** *Bacillus subtilis*, bacterial nutrient utilisation, cysteine synthesis, *Escherichia coli*, plant‐derived environments, sulphur acquisition pathway

## Abstract

Bacteria and plants are closely associated with human society, in fields such as agriculture, public health, the food industry, and waste disposal. Bacteria have evolved nutrient‐utilisation systems adapted to achieve the most efficient growth in their major habitats. However, empirical evidence to support the significance of bacterial nutrient utilisation in adaptation to plants is limited. Therefore, we investigated the genetic and nutritional factors required for bacterial growth in plant extracts by screening an *Escherichia coli* gene‐knockout library in vegetable‐based medium. Mutants lacking genes involved in sulphur assimilation, whereby sulphur is transferred from sulphate to cysteine, exhibited negligible growth in vegetable‐based medium or plant extracts, owing to the low cysteine levels. The reverse transsulphuration pathway from methionine, another pathway for donating sulphur to cysteine, occurring in bacteria such as *Bacillus subtilis* , also played an important role in growth in plant extracts. These two sulphur‐assimilation pathways were more frequently observed in plant‐associated than in animal‐associated bacteria. Sulphur‐acquisition pathways for cysteine synthesis thus play a key role in bacterial growth in plant‐derived environments such as plant residues and plant exudates.

## Graphical abstract

Plant extracts are characterised by low levels of free cysteine (Cys). Bacterial uptake of glutathione (GSH), a cysteine‐containing tripeptide, is inhibited by the presence of other amino acids (AAs). Thus, the cysteine synthesis pathway through sulphate (SO_4_^2−^) acquisition is necessary for growth in plant exudates and residues.

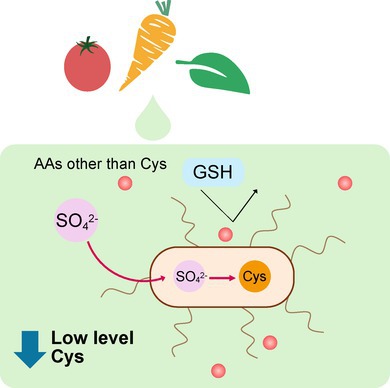

## 1 Introduction

Bacterial species exhibit different nutrient‐utilisation capabilities depending on their genetic background. Bacteria exhibit preferences for sugar and amino acids (AAs), and these preferences have been examined primarily with respect to the composition of the culture media and as phenotypes for taxonomic characterisation (Liu et al. 2020). Although evolutionary adaptations in nutrient utilisation have enabled bacteria to grow efficiently in their major habitats, empirical evidence on the significance of bacterial nutrient utilisation in environmental adaptation is limited.

Bacteria participate directly in plant growth by causing diseases and by communicating with plants in the rhizosphere. Bacteria and plants are closely associated with human society, particularly in areas such as public health (Fatima et al. 2025), the food industry (Swain et al. 2014), and waste disposal (Pan et al. 2012). For example, food poisoning, such as that caused by eating raw or processed vegetables contaminated with *Escherichia coli* or *Bacillus cereus* , presents an important public health problem (Fatima et al. 2025). Pickled vegetables that are fermented mainly with *Lactobacillaceae* bacteria are produced and consumed worldwide (Swain et al. 2014; Alameri et al. 2022). In recent years, in order to reduce the environmental impact of the disposal of plant‐based biogenic waste, composting and biofuel production using bacteria including *Bacillus*, *Pseudomonas*, and *E. coli* have been promoted (Pan et al. 2012). Considering these factors, it is therefore crucial to identify the nutritional factors underlying bacterial growth in plant residues and plant extracts. As plants exhibit characteristic carbohydrate and AA compositions that differ from those of animals and other types of matter, plant‐specific nutritional factors affecting bacterial growth are likely to exist.

Cysteine, which contains sulphur in its thiol group, is important in sensing redox states and maintaining the three‐dimensional structure of proteins via disulphide bonds. In bacteria, the sulphur atom of cysteine is donated by sulphates, thiosulphates, sulphonates, sulphides, and methionine. To incorporate this sulphur atom, oxidised sulphur compounds must be reduced to sulphides, which is energetically costly, particularly under aerobic conditions, and is therefore subject to selective pressure (Sekowska et al. 2000). *Bacillus subtilis* uses sulphates, thiosulphates, sulphonates, sulphides, and methionine as sulphur sources; *Escherichia coli* utilises the first four of these sulphur sources, but not methionine (Lithgow et al. 2004; Hullo et al. 2007); and *Staphylococcus aureus* utilises only thiosulphates and sulphides (Lithgow et al. 2004). In bacteria that cannot synthesise cysteine from methionine, sulphur atoms are unidirectionally transferred from cysteine to methionine, increasing the requirement for cysteine. Conversely, bacteria that can transfer sulphur atoms bidirectionally between methionine and cysteine can effectively tolerate cysteine‐limited environments, as they can obtain sulphur from methionine during sulphate starvation (Seiflein and Lawrence 2006). The enrichment of pathways whereby sulphur is provided to cysteine is considered essential for bacterial growth in oligotrophic environments (such as in natural waters) but optional in AA‐rich environments (such as host tissue).

Nutrient composition varies depending on plant species. To identify the common factors required for bacterial growth in plant extracts, we selected mixed vegetable extracts. V8 juice (Campbell Soup Company, NJ, USA), a 100% vegetable juice comprising tomato, carrot, celery, beet, parsley, lettuce, watercress, and spinach (Mycological Society of America and Stevens 1974), contains only plant‐derived substances, according to the manufacturer's food composition table. We therefore used a V8‐based medium for knockout screening of *E. coli* . The findings suggest that sulphur acquisition for cysteine synthesis was important for growth in V8‐based medium. The importance of sulphur acquisition was similar for growth on most of the vegetable and wild grass extracts that we tested. These findings clarify the nutritional factors necessary for bacterial growth in plant‐derived environments.

## 2 Experimental Procedures

### 2.1 Bacteria and Culture Media

*Escherichia coli* BW25113, the Keio collection gene knockout library, and *Lactococcus lactis* subsp. *lactis* (NBRC12007) were provided by the Japan National Bioresource Project (NBRP). *Bacillus subtilis* 168 (BGSC_168) was provided by the *Bacillus* Genetic Stock Center (BGSC) and the gene‐deletion mutants (BsuΔ*cysC*, BKE15600; BsuΔ*cysE*, BKE00930; and BsuΔ*mccA*, BKE27260) were provided by NBRP *B. subtilis* . *Pectobacterium carotovorum* subsp. *carotovorum* MAF301049 and *Xanthomonas campestris* pv. *campestris* MAFF211374 were provided by the Japan National Agriculture and Food Research Organization (NARO). The *E. coli* O157:H7 Sakai, *S. aureus* RN4220, and *S. aureus* Newman strains were reported in previous papers (Lorenz and Duthie 1952; Peng et al. 1988; Hayashi 2001).

The following culture media were used: lysogeny broth (LB) medium (1% [w/v] tryptone [Nacalai Tesque, Kyoto, Japan], 0.5% [w/v] yeast extract [Nacalai Tesque], and 1% [w/v] NaCl [Nacalai Tesque]); foetal bovine serum (FBS) (Nichirei Biosciences, Tokyo, Japan; Cat. No. 175012, Lot No. 18M00F); M9 medium (M9 minimal medium (standard) 2010); and Spizizen minimal medium (SP) (0.2% [w/v] (NH_4_)_2_SO_4_ [Nacalai Tesque], 1.4% [w/v] K_2_HPO_4_ [Nacalai Tesque], 0.6% [w/v] KH_2_PO_4_ [Nacalai Tesque], 1% [w/v] sodium citrate [Wako Chemical, Osaka, Japan], 5% [w/v] glucose [Wako Chemical], 0.1% [w/v] MgSO_4_ [Sigma‐Aldrich, St Louis, MO, USA], and 50 μg/mL tryptophan [Wako Chemical]) (Anagnostopoulos and Spizizen 1961). FBS was inactivated at 55°C for 30 min before use. To prepare the V8 medium, 340 mL of V8 juice (Campbell Soup Company, NJ, USA) was stirred with 5 g of CaCO_3_ for 15 min to adjust pH and centrifuged at 2150 *g* for 15 min. The supernatant was five‐fold diluted with ultrapure water and autoclaved. Vegetables were purchased at a supermarket, and the leaves of mugwort and clover were collected on the Tsushima campus of Okayama University. The vegetables and plants were ground with a blender or grater. The homogenates were filtered with polyethylene terephthalate nonwoven fabric and centrifuged at 21,500 *g* for 15 min. The supernatants were filtered through a 0.22 μm cartridge filter and used for bacterial culture.

### 2.2 Culture Conditions

The bacteria were precultured at 30°C for 18 h in 50 mL polypropylene tubes containing 5 mL LB medium with shaking (for all bacteria except *L. lactis*) or without shaking (for *L. lactis* ). For each bacterial preculture, 1 μL was inoculated into 100 μL of LB, V8 medium, or FBS at 30°C in 96‐well flat‐bottom plates. To determine the requirement of cysteine, cystine, glutathione, and methionine, the bacteria precultures were centrifuged at 4000 *g* for 5 min, washed with 5 mL saline to remove any remaining LB medium, and resuspended to approximately 1 × 10^9^ cfu/mL in saline. The resuspended bacterial cells were used as inocula, as described above. The following reagents were used: l‐cysteine (Wako, #039‐20652), l‐cystine (Sigma‐Aldrich, #C8755), glutathione (in reduced form) (Nacalai, #08786‐32), and l‐methionine (Wako, #133‐01602).

### 2.3 Gene Knockout Library Screening Using V8 Medium

The *E. coli* Keio collection (Baba et al. 2006) was precultured at 37°C for 18 h in 100 μL of LB medium in 96‐well flat‐bottom plates. Of each bacterial preculture, 1 μL was inoculated into 100 μL of LB or V8 medium at 37°C in 96‐well flat‐bottom plates, and the increase in OD_595_ (ΔOD_595_) was measured after 3 h. The experiment was performed twice, and the average value was used for analysis. Mutants that scored ΔOD_595_ values in LB < 0.1 were excluded from the analysis. Gene Ontology (GO) enrichment analysis was performed using ShinyGO 0.80 online software (http://bioinformatics.sdstate.edu/go80/) (Ge et al. 2020).

### 2.4 Gene Disruption

As gene‐deficient mutants of *E. coli* BW25113, strains in the Keio collection were used after confirming gene deletion by PCR (Figure S1A). As an exception, we created a *cysB*‐deficient mutant because *cysB* deletion and the cysteine‐auxotrophic phenotype of JW1267‐KC in the Keio collection were not confirmed (Figure S1B). The one‐step inactivation method (Datsenko and Wanner 2000) was used to delete *cysB* from *E. coli* BW25113. The PCR fragments, amplified using oligonucleotides #15 and #16 (Table S1) and pKD13 as a template, were electroporated into the BW25113 strain carrying pKD46 expressing the phage λ Red recombinase, and a *cysB*‐deficient mutant was selected by culturing the electroporated cells at 30°C on LB agar with 100 μg/mL kanamycin. Strains in which the *cysB* open reading frame (ORF) was replaced with the kanamycin‐resistance gene were confirmed by PCR‐based genotyping (Figure S1C), cultured at 43°C to remove pKD46, and those strains that had lost ampicillin resistance were used as Δ*cysB* mutants. For *B. subtilis* 168, the *cysC* and *cysE* mutants (BKE15600 and BKE00930) were used after genotyping by PCR (Figure S1D). For the *mccAB* operon deletion mutant (Δ*mccAB*), the fragment containing the erythromycin cassette inserted into the *mccA* locus and 1 kbp upstream of the *mccA* locus was PCR‐amplified using oligonucleotides #23 and #24 (Table S1) and the genomic DNA of the Δ*mccA*. The 1 kbp DNA fragment downstream of *mccB* was PCR‐amplified using oligonucleotides #25 and #26 (Table S1) and the genomic DNA of the strain 168. The two PCR products were joined using recombinant PCR and used to transform strain 168 via natural competence, as described previously (Ishikawa et al. 2022). To construct Δ*cysC*Δ*mccAB*, the genomic DNA of Δ*mccAB* was introduced to Δ*cysC* whose erythromycin‐resistance cassette was removed using pDR244 (Koo et al. 2017).

### 2.5 Plasmid Construction and Transformation

Genomic DNA of BW25113 was extracted using a QIAamp DNA blood Mini Kit (Qiagen, Hilden, Germany). DNA fragments containing the *cysC*, *cysE*, or *cysH* genes, with the Shine‐Dalgarno sequence, were amplified by PCR using BW25113 genomic DNA as a template and the oligonucleotides listed in Table S1. The amplified DNA fragments were inserted into the EcoRI and HindIII sites in the pMW118 vector, thus generating pMW118‐cysC, pMW118‐cysE, and pMW118‐cysH. pMW118‐cysC, pMW118‐cysE, pMW118‐cysH, or pKD46 (Datsenko and Wanner 2000) were introduced into *E. coli* BW25113 or its gene‐deletion mutants by electroporation.

### 2.6 Measurements of Cysteine Content

LB medium, V8 medium, FBS, or vegetable extracts were mixed with ethanol to a final concentration of 70%, and the proteins were precipitated by centrifugation at 21,400 *g* for 15 min. The supernatant was dried using an evaporator and dissolved in 10 mM HEPES–KOH buffer (pH 6.8). The amino acid–peptide solution was reduced by adding 1/4 the volume of tris (2‐carboxyethyl) phosphine (TCEP), immobilised on agarose CL‐4B (Sigma‐Aldrich) at room temperature for 2 h, after which the TCEP‐beads were removed using a filter column. The eluates were treated with a MicroMolar Cysteine Assay Kit (ProFoldin, Hudson, MA, USA) and measured on a Fluoroskan Ascent CF microplate fluorometer (Thermo Fisher Scientific, Waltham, MA, USA) at excitation 485 nm and emission 527 nm, according to the manufacturer's instructions.

### 2.7 Amino‐Acid Quantification by High Performance Liquid Chromatography

Precolumn labelling with amine‐reactive 4‐fluoro‐7‐nitro‐2,1,3‐benzoxadizole (NBD‐F) was performed according to the methods of Hattori et al. (2017). Proteins were removed using 45% methanol/acetonitrile.

### 2.8 Metabolic Pathway Analysis

The presence or absence of metabolic pathways in the bacteria was investigated using Kyoto Encyclopedia of Genes and Genomes (KEGG) (https://www.kegg.jp) (Kanehisa et al. 2023). The isolation source of each bacterial species was referenced in the Bac*Dive* data base (https://bacdive.dsmz.de) (Reimer et al. 2022). When multiple accessions for a single bacterial species were identified, the one with the earliest ID was extracted. The following mammal‐related source tags in *Bac*Dive were used: Disease, Fluids, Gastrointestinal tract, Human, Mammals, Oral cavity and Airways, Organ, Patient, and Urogenital tract. As plant‐related source tags, we used ‘Plant’ and ‘Plant infections.’ Bacterial metabolic pathway distribution maps were created using Microsoft Excel and heatmaps using GraphPad Prism 9.

## 3 Results

### 3.1 Bacteria Commonly Found in Plants Can Grow Effectively on V8 Medium

**FIGURE 1:**
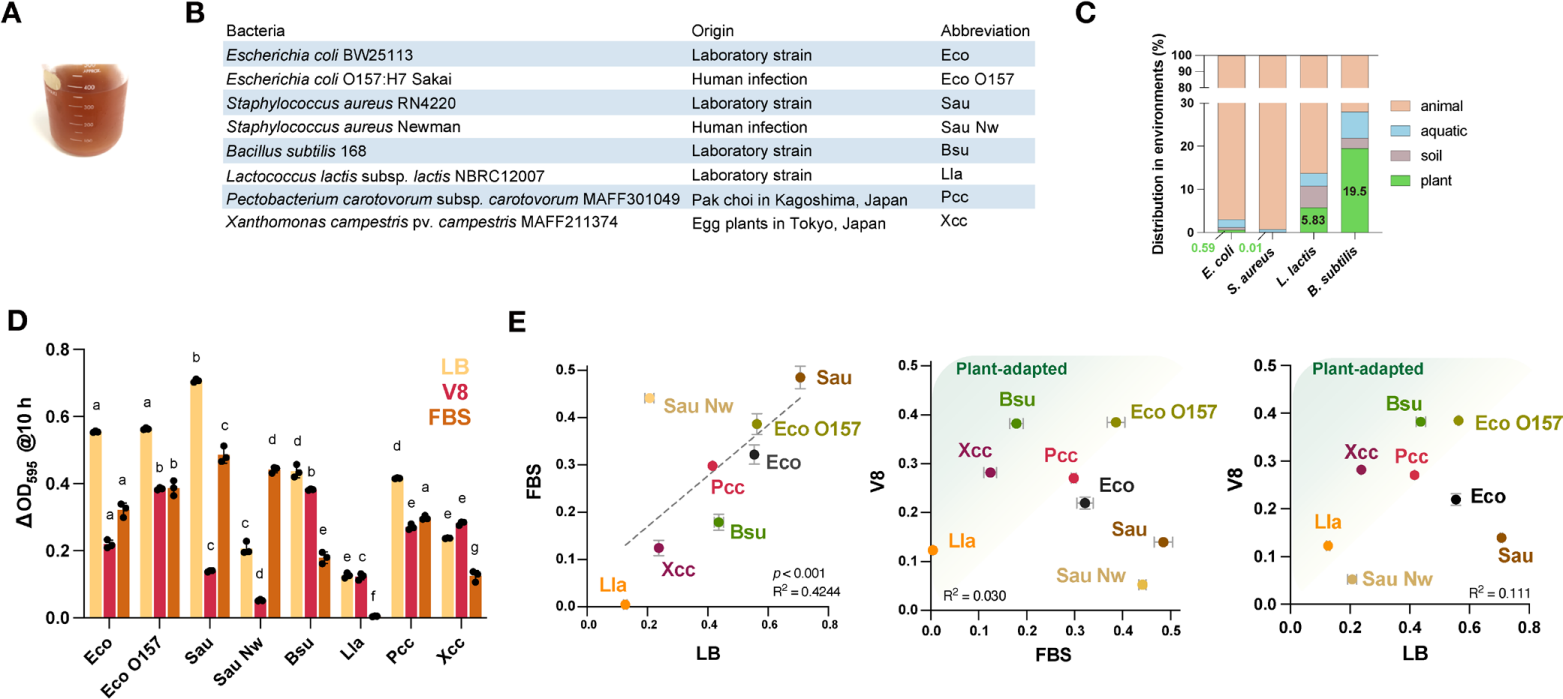
Plant‐adapted bacteria exhibit higher growth rate on modified V8 medium. (A) V8 medium. (B) Bacteria used in this paper. (C) Distribution of *Escherichia coli* , *Staphylococcus aureus* , *Lactococcus lactis* , and *Bacillus subtilis* in the natural environment. The percentages of 16S rRNA gene sequence reads in the metagenomes for each type of environment were analysed using the Microbe Atlas database (https://microbeatlas.org). (D) Growth of bacteria in lysogeny broth (LB), V8 medium, and foetal bovine serum (FBS). Abbreviations for the bacteria are listed in (B). The increase in OD_595_ (ΔOD_595_) after 10 h incubation at 30°C is indicated. Data are shown as means ± SD; different letters indicate significant differences within the same medium, *n* = 3, *p* < 0.05, using Tukey's multiple comparisons test. (E) Plot of ΔOD_595_ after culturing bacteria in LB, V8 medium, and FBS at 30°C for 10 h. Data are shown as means ± SD. *R*‐squared values from simple regression are shown. Lines of best approximation and significance levels are shown for FBS and LB.

We prepared V8‐based medium by adding CaCO_3_ to V8 juice to adjust the pH, without further supplementation (Figure 1A). We collected the following bacteria, which exhibit different levels of association with plants: *E. coli* BW25113 (Datsenko and Wanner 2000), enterohemorrhagic *E. coli* O157:H7 Sakai (Hayashi 2001), *Staphylococcus aureus* RN4220 (Peng et al. 1988), *S. aureus* Newman (Lorenz and Duthie 1952), *Lactococcus lactis* subsp. *lactis* (Hirsch 1951), *Bacillus subtilis* (Burkholder and Giles 1947), and two plant pathogenic bacteria, *Pectobacterium carotovorum* subsp. *carotovorum* and *Xanthomonas campestris* pv. *campestris* (Figure 1B). Compared with *S. aureus* , *E. coli* is more likely to be found in plant‐associated environments (Figure 1C), consistent with *E. coli* and enterohemorrhagic *E. coli* frequently contaminating vegetables (Michino et al. 1999). *Bacillus subtilis* and *L. lactis* are frequently found plant‐associated environments (Figure 1C), consistent with their frequent detection on plants (Yu et al. 2020; Soto‐Giron et al. 2021).

The bacteria were cultured aerobically in LB medium, V8 medium, or FBS (Figure 1D). The growth of the bacteria in LB medium and FBS, indicated by the increase in optical density at 595 nm (ΔOD_595_) after 3 h of incubation, was highly correlated (*R* ^2^, 0.4244), although that of *S. aureus* Newman differed between LB medium and FBS (Figure 1E). In contrast, no correlation was observed between bacterial growth in V8 medium and in FBS or LB medium (Figure 1E). *Bacillus subtilis* , *X. campestris* , and *L. lactis* , which are common in plants, grew well in V8 medium, whereas *S. aureus* RN4220 and *E. coli* BW25113, which occur in animals, grew relatively well in FBS and LB medium (Figure 1E). The nutrient compositions of LB medium, V8 medium, and FBS are presented in Figure S2. V8 medium is sugar‐rich and contains moderate levels of free AAs, more than half of which are glutamic acid, aspartic acid, and asparagine.

### 3.2 Sulphur Assimilation Into Cysteine Is Essential for *E. coli* Growth in V8‐Based Medium

**FIGURE 2:**
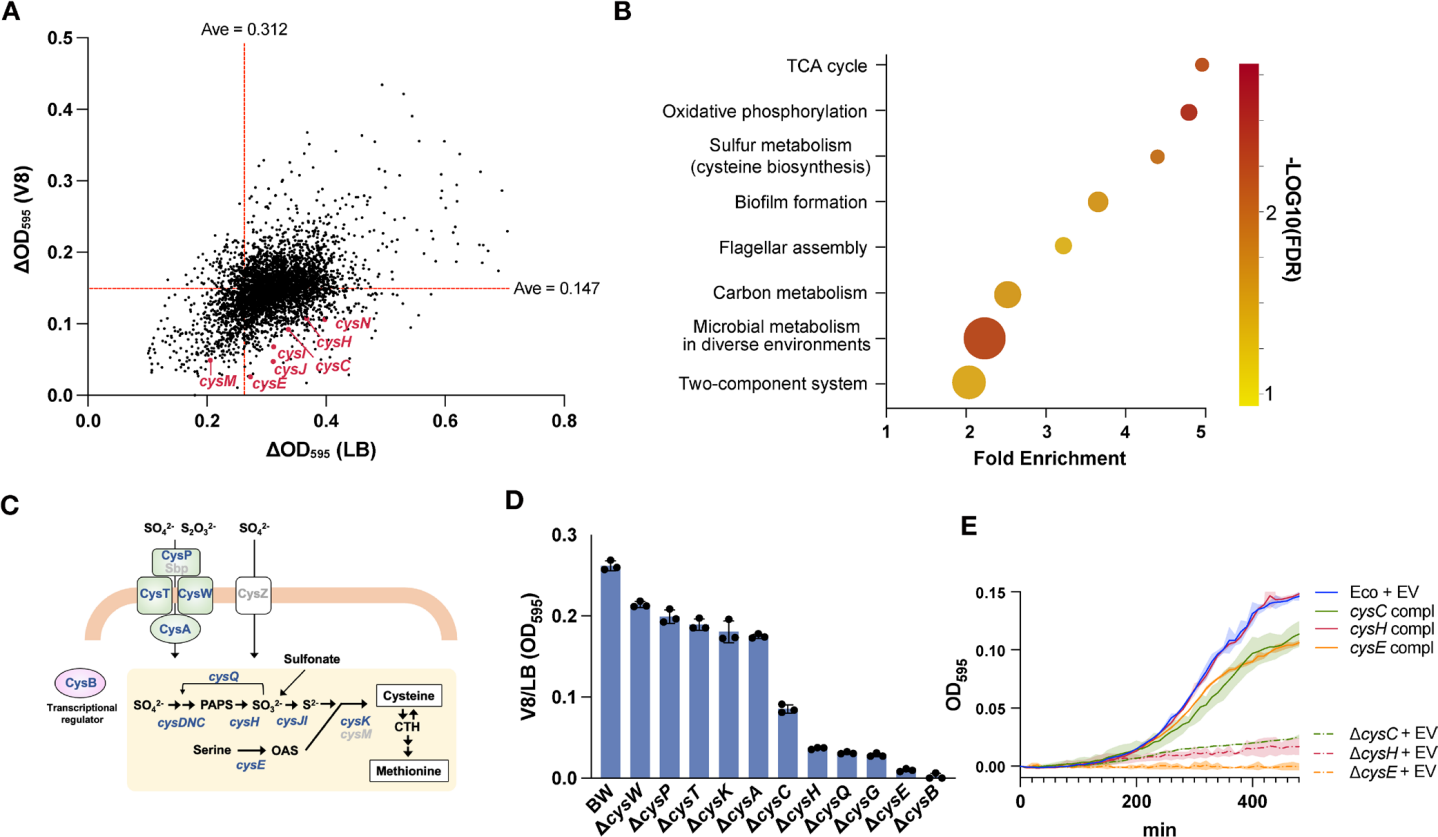
Screening of the *E. coli* gene knockout library using V8 medium. (A) Output of *E. coli* BW25113 screening using the Keio collection, presenting the average ΔOD_595_ following incubation in duplicate at 37°C for 3 h in LB (horizontal axes) and V8 medium (vertical axes). Mutants with ΔOD_595_ < 0.1 in LB were excluded from the analysis. The red lines show the average growth of all of the mutants in LB and V8 medium. The red points indicates cysteine‐auxotrophs whose V8/LB ΔOD_595_ (ΔOD_595_ on V8 medium divided by that on LB medium) values were in the lowest 5%. (B) Gene Ontology (GO) enrichment analysis of genes knocked out in mutants with reduced growth in V8 medium. Mutants with V8/LB ΔOD_595_ values in the lowest 5% were used for analysis. E‐ratio, circle size, and circle colour indicate fold enrichment, the number of genes, and raw *p*‐value, respectively. (C) Schematic of the cysteine biosynthetic pathway of *E. coli* BW25113. The genes used in (D) are shown in blue. (D) V8/LB ΔOD_595_ values of cysteine‐auxotrophic mutants grown at 37°C for 3 h. Data are shown as means ± SD, *n* = 3. (E) Complementation experiment of cysteine‐auxotrophic mutants. The growth of the Δ*cysC*, Δ*cysE*, and Δ*cysH* mutants carrying an empty vector pMW118 (EV) (Δ*cysC* + EV, Δ*cysE* + EV, Δ*cysH* + EV) or complemented (‘compl’) with pMW118‐cysC, ‐cysE, and ‐cysH (*cysC* compl, *cysE* compl, *cysH* compl) was compared with that of the parent strain ( *E. coli* BW25113) carrying the empty vector pMW118 (Eco + EV). Coloured lines and areas show the mean and standard deviations (±SD), respectively, for Eco + EV (solid blue line), Δ*cysC* + EV (dotted green line), *cysC* compl (solid green line), Δ*cysE* + EV (dotted orange line), *cysE* compl (solid orange line), Δ*cysH* + EV (dotted red line), and *cysH* compl (solid red line). *n* = 3.

To investigate the functions of the genes required for bacterial growth in plant extracts, we screened an *E. coli* BW25113 gene‐knockout mutant library (Baba et al. 2006) in V8 medium (Figure 2A). To identify genes important for growth in V8 medium, we performed Gene Ontology analysis of the 192 genes deleted in those mutants with V8/LB ΔOD_595_ (ΔOD_595_ following incubation on V8 medium divided by that following incubation on LB medium) in the lowest 5%. Pathways involved in energy production, including the tricarboxylic acid cycle, oxidative phosphorylation, and carbon metabolism, were significantly enriched in these mutants, indicating a difference in the primary carbon source in LB and V8 media (Figure 2B; Table S2). Notably, the sulphur‐assimilation pathway was significantly enriched, which means that the sulphur source was limited in V8 medium. In *E. coli* , this pathway overlaps with the pathway of cysteine biosynthesis from sulphate (Figure 2C).

The V8/LB ΔOD_595_ values of the cysteine‐auxotrophic *E. coli* mutants were significantly lower than those of the parent strain (Figure 2D; Figure S3). Sulphate‐transporter component mutants (*cysA*/*cysP*/*cysT*/*cysW*) exhibited better growth than the other mutants, likely owing to their redundant functions with *cysZ*; conversely, mutants of genes responsible for enzymatic reactions in the cysteine biosynthetic pathway (*cysC*/*cysH*/*cysQ*/*cysG*/*cysE*) and in transcription factor *cysB*, which controls the transcription of genes involved in cysteine biosynthesis, exhibited worse growth. In V8 medium, complementation of *cysC*, *cysE*, and *cysH* restored the growth of the Δ*cysC*, Δ*cysE*, and Δ*cysH* mutants, respectively (Figure 2E).

### 3.3 V8 Medium Provides Insufficient Cysteine for *E. coli* Growth

**FIGURE 3:**
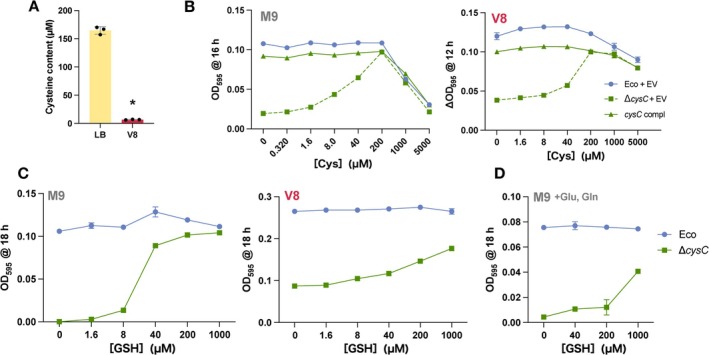
Cysteine available to bacteria is limited in plant extracts. (A) Quantification of cysteine contents in LB and V8 medium. Data are shown as means ± SD. Asterisks indicate significant differences, *n* = 3, *p* < 0.05, using Student's *t*‐test. (B) Growth of *E. coli* BW25113 in M9 and V8 media supplemented with free cysteine. ΔOD_595_ values following 16 h (for M9) or 12 h (for V8) of incubation at 30°C are plotted for BW25113 and Δ*cysC* harbouring pMW118 (Eco + empty vector pMW118 (EV), solid line with blue circles; Δ*cysC* + EV, dotted line with green squares), and Δ*cysC* harbouring pMW118‐cysC (*cysC* compl, solid line with green triangles). Data are shown as means ± SD, *n* = 4. (C) Growth of *E. coli* BW25113 in M9 (left) and V8 media (right) supplemented with reduced glutathione (GSH). ΔOD_595_ values following 18 h of incubation at 30°C are plotted for *E. coli* BW25113 (Eco, solid line with blue circles) and Δ*cysC* (solid line with green squares). (D) GSH utilisation in the presence of glutamate and glutamine. ΔOD_595_ values following incubation at 18 h at 30°C in M9 medium supplemented with 5 mM glutamate and 5 mM glutamine are plotted for *E. coli* BW25113 (Eco, solid line with blue circles) and Δ*cysC* (solid line with green squares). Data are shown as means ± SD; *n* = 3.

Based on our finding that *E. coli* that lacks cysteine biosynthesis, exhibited reduced growth in V8 medium, we speculated that V8 medium has low content of cysteine and cystine, a cysteine dimer bound by a disulphide bond. However, the instability of cysteine precludes its detection via high performance liquid chromatography (Figure S2B). Therefore, the free‐AA containing fraction was treated with a reducing agent to convert cystine to cysteine, which was then quantified using a fluorescent dye that is highly reactive to cysteine. The cysteine concentration of LB medium was 164 μM, whereas that of V8 medium was ca. 1/25 of that, at 6.75 μM (Figure 3A).

We next quantified the cysteine concentration required to restore Δ*cysC* mutant growth in M9 minimal medium. Adding low concentrations of cysteine gradually restored Δ*cysC* mutant growth; the maximum growth, comparable to that of the parent strain, was achieved at 200 μM cysteine (Figure 3B). Adding ≥ 1 mM cysteine inhibited growth in all strains, consistent with prior findings (Harris 1981; Korshunov et al. 2020). Cystine similarly restored Δ*cysC* mutant growth, with no inhibition occurring at high concentrations (Figure S4). In V8 medium, the Δ*cysC* mutant exhibited maximum growth at 200 μM cysteine, as in M9 medium (Figure 3B). Therefore, cysteine at ca. 200 μM is required to fully restore the growth of cysteine‐auxotrophs, with these strains exhibiting severe growth defects in V8 medium, owing to the lack of cysteine sources.

Plant tissue is widely estimated to contain glutathione at tens to hundreds of micromoles per litre (Pivato et al. 2014). Although many bacteria, including *E. coli* , can utilise glutathione as a cysteine source (Suzuki et al. 1993, 2005; Sherrill and Fahey 1998; Minami et al. 2004; Alkhuder et al. 2009), *E. coli* BW25113 was apparently unable to utilise glutathione as a cysteine source in V8 medium (Figure 2E). Therefore, we added reduced glutathione (GSH) to the M9 and V8 media to examine Δ*cysC* mutant growth. Adding 40 μM GSH to M9 medium significantly restored Δ*cysC* mutant growth; adding 200 μM GSH resulted in growth almost equivalent to that of the parent strain (Figure 3C). In contrast, in V8 medium, even with the addition of 200 μM GSH, Δ*cysC* mutant growth recovered only weakly; even with the addition of 1 mM GSH, it did not reach that of the parent strain (Figure 3C), potentially owing to the presence of other AAs that inhibited GSH uptake or degradation. We then added 5 mM each of glutamine and glutamic acid, which are abundant in many plants (Figure S5), to M9 medium; the efficiency of GSH utilisation by the Δ*cysC* mutant was clearly reduced, and its growth remained below that of the parental strain, even with the addition of 1 mM GSH, as in V8 (Figure 3D).

### 3.4 Sulphur Assimilation Is Essential for *E. coli* Growth in Plant Extracts

**FIGURE 4:**
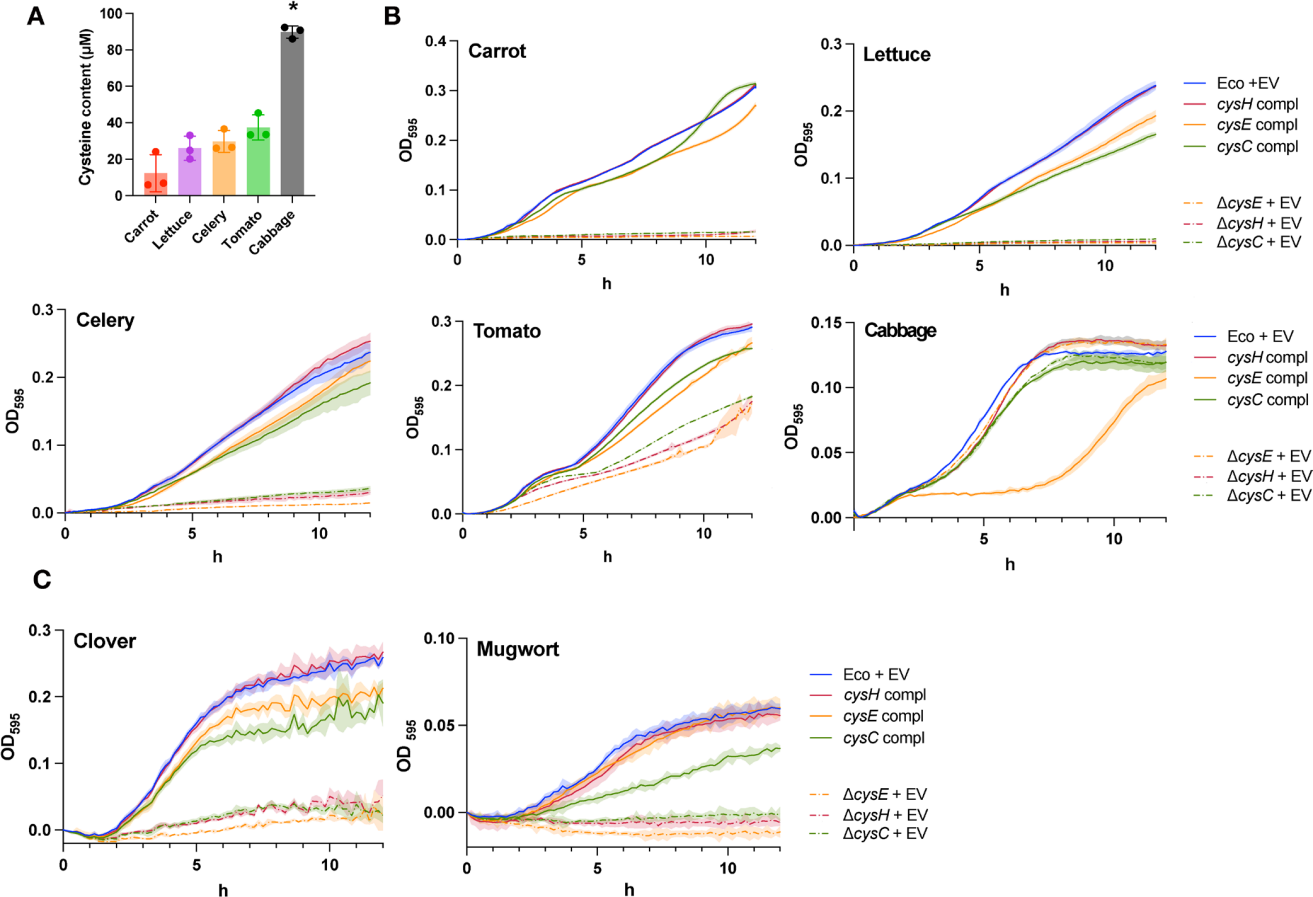
Sulphur assimilation is essential for *E. coli* growth in vegetables. (A) Quantification of cysteine contents in vegetables. Data are shown as means ± SD. Asterisks indicate significant differences, *n* = 3, *p* < 0.05, using Tukey's multiple comparisons test. (B, C) Growth of *E. coli* BW25113 (Eco) + empty vector (EV), Δ*cysC* + EV, Δ*cysE* + EV, Δ*cysH* + EV and of the complemented (‘compl’) forms (*cysC* compl, *cysE* compl, and *cysH* compl) in vegetable and plant extracts. Tomato extract was adjusted to pH 7.0. Coloured lines and areas show the mean and standard deviations (±SD), respectively, for Eco + EV (solid blue line), Δ*cysC* + (dotted green line), *cysC* compl (solid green line), Δ*cysE* + EV (dotted orange line), *cysE* compl (solid orange line), Δ*cysH* + EV (dotted red line), and *cysH* compl (solid red line). *n* = 3.

We examined whether cysteine‐biosynthesis‐defective *E. coli* mutants exhibited defective growth in specific vegetable extracts. First, the free AA content of carrot, lettuce, celery, tomato, and cabbage was examined. Similar to V8 medium, these extracts were rich in polar AAs such as aspartic acid and asparagine (Figure S5). The cysteine contents of the carrot, lettuce, celery, tomato, and cabbage extracts were 12.3, 26.0, 29.7, 37.4, and 89.7 μM (Figure 4A), and their pH values were 6.45, 6.43, 6.23, 4.37, and 6.37, respectively. The pH of the tomato extract was adjusted to 7.0 because it was outside the optimum range for bacterial growth. In carrot, lettuce, and celery, which have low cysteine contents, the Δ*cysC*, Δ*cysE*, and Δ*cysH* mutants did not grow, whereas the complemented strains exhibited growth comparable to that of the parent strain (Figure 4B). In tomato, the Δ*cysC*, Δ*cysE*, and Δ*cysH* mutants showed weaker growth than the complemented and parental strains. In cabbage, which exhibited relatively high cysteine and cystine content among the vegetables examined, the Δ*cysC* and Δ*cysH* mutants showed comparable growth to their complemented and parent strains, potentially because *E. coli* can utilise S‐methyl‐l‐cysteine, an S‐methylated derivative of cysteine abundant in Brassicaceae (Coode‐Bate et al. 2019), for cysteine biosynthesis (Ishiwata et al. 1989). We also examined the growth of these bacterial strains in plants other than vegetables. In the extracts of clover and mugwort, the Δ*cysC*, Δ*cysE*, and Δ*cysH* mutants exhibited significantly lower growth than the parent strain (Figure 4C).

### 3.5 Bacterial Growth in Plant Extracts Is Affected by Differences in Sulphur Acquisition Pathways for Cysteine Synthesis

**FIGURE 5:**
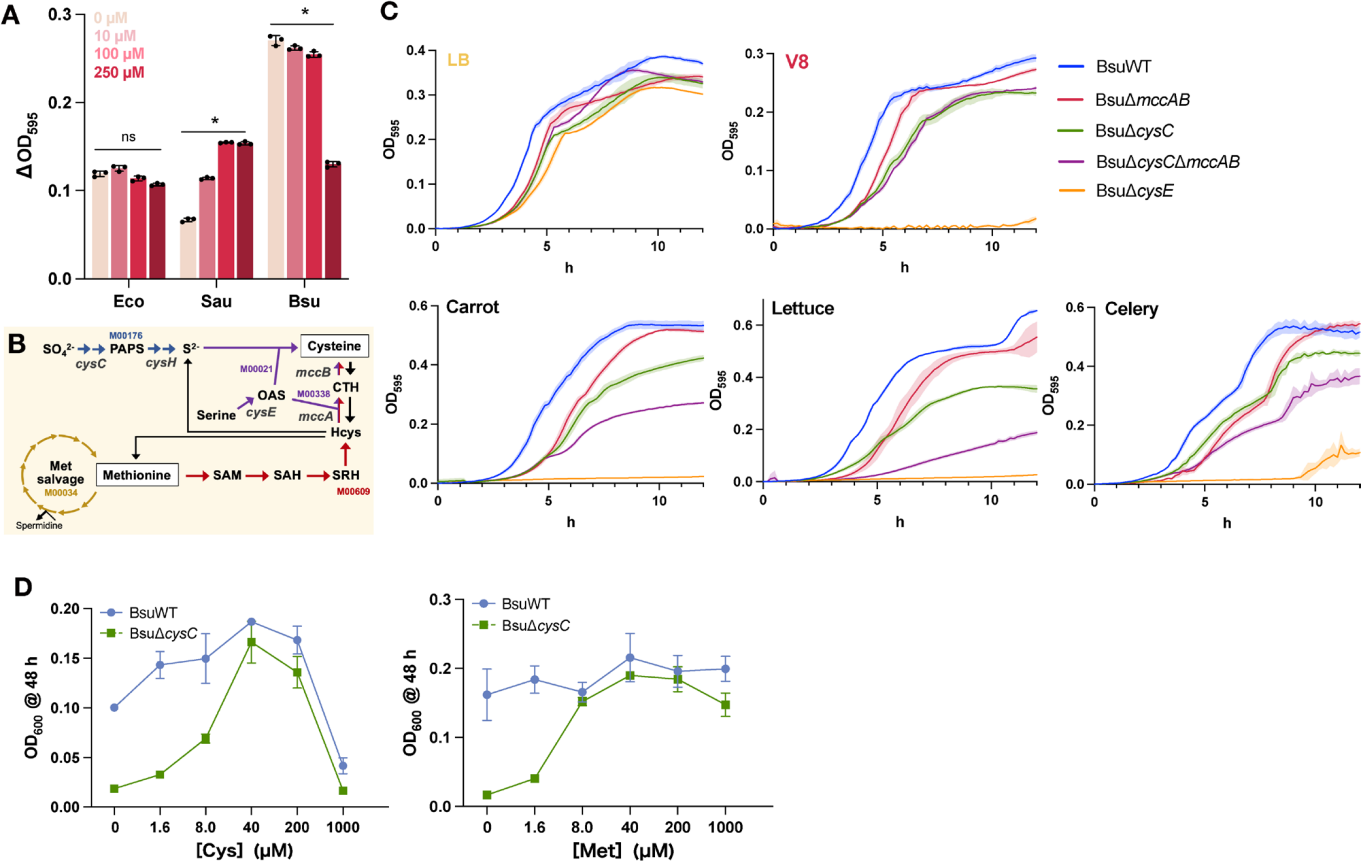
Sulphur acquisition from sulphate and methionine is necessary for *B. subtilis* growth in plant extracts. (A) *Escherichia coli* BW25113 (Eco), *Staphylococcus aureus* RN4220 (Sau), and *Bacillus subtilis* (Bsu) growth in V8 medium supplemented with cysteine. ΔOD_595_ values after 14 h of incubation at 30°C is shown. Data are shown as means ± SD; asterisks indicate significant differences, *n* = 3, *p* < 0.05, using Dunnett's multiple comparisons test. (B) Cysteine biosynthesis pathway from sulphate ions or methionine and the methionine salvage pathway in Bsu. PAPS, Phosphoadenosine 5′‐phosphosulphate; SAM, S‐adenosyl‐l‐methionine; SAH, S‐adenosyl‐l‐homocysteine; SRH, S‐ribosylhomocysteine; OAS, O‐Acetyl‐l‐serine; Hcys, homocysteine; CTH, cystathionine. The KEGG module numbers are given and each pathway is colour‐coded. (C) Growth of BsuΔ*cysC*, BsuΔ*cysE*, BsuΔ*mccAB*, and BsuΔ*cysC*Δ*mccAB* at 30°C in LB medium, V8 medium, and vegetable extracts, relative to that of the parent strain (BsuWT). Coloured lines and areas show the mean and standard deviations (±SD). Respectively, for BsuWT (solid blue line), BsuΔ*cysC* (solid green line), BsuΔ*cysE* (solid orange line), BsuΔ*mccAB* (solid red line), and BsuΔ*cysC*Δ*mccAB* (solid purple line). *n* = 3. (D) Growth of Bsu in Spizizen minimal medium agar (SP) medium supplemented with cysteine (Cys) or methionine (Met). ΔOD_595_ values following incubation at 48 h at 30°C are plotted for BsuWT (solid line with blue circles) and BsuΔ*cysC* (solid line with green squares). Data are shown as means ± SD, *n* = 3.

We added cysteine to V8 medium and examined the growth of *E. coli* BW25113, *S. aureus* RN4220, and *B. subtilis* . The addition of 100 μM cysteine did not significantly alter the growth of *E. coli* BW25113 (Figure 5A) but significantly improved that of *S. aureus* RN4220, while reducing that of *B. subtilis* . Adding 250 μM cysteine strongly and more significantly inhibited *B. subtilis* growth. *Staphylococcus aureus* lacks the sulphate‐assimilation pathway, suggesting that it cannot obtain sufficient cysteine for growth from V8 medium. *Bacillus subtilis* has a pathway for cysteine synthesis from methionine via reverse transsulphuration in addition to sulphate‐assimilation pathway (Figure 5B). Methionine is present in vegetables at approximately 10 μM (Figure S5), potentially accounting for the superior growth of *B. subtilis* compared to *E. coli* in the cysteine‐poor V8 medium.

To verify this, we established *B. subtilis* mutants lacking *cysC* (BsuΔ*cysC*), *cysE* (BsuΔ*cysE*), the *mccAB* operon (BsuΔ*mccAB*), and both *cysC* and *mccAB* operons (BsuΔ*cysC*Δ*mccAB*). BsuΔ*cysC* can synthesise cysteine from methionine, whereas BsuΔ*cysE* cannot synthesise it because it lacks O‐acetyl‐l‐serine, which is required for cysteine synthesis from both sulphate and methionine (Figure 5B; Figure S6). Similarly, BsuΔ*cysC*Δ*mccAB* cannot synthesise cysteine from sulphate and methionine, as it lacks the sulphur assimilation and reverse transsulphuration pathways. In LB medium, these *B. subtilis* mutants (BsuΔ*cysC*, BsuΔ*cysE*, BsuΔ*mccAB*, and BsuΔ*cysC*Δ*mccAB*) exhibited growth comparable to that of wild‐type (WT) *B. subtilis* (BsuWT). In V8 medium, all the *B. subtilis* strains exhibited good growth, except for BsuΔ*cysE*, which exhibited negligible growth (Figure 5C) owing to lack of cysteine in the V8 medium. In the carrot, lettuce, and celery extracts, BsuΔ*cysC* showed slightly worse growth than BsuWT (Figure 5C). The stationary‐phase turbidity of BsuΔ*mccAB* was comparable to that of the BsuWT, albeit with a slightly extended lag time (Figure 5C). Notably, BsuΔ*cysC*Δ*mccAB* exhibited markedly worse growth than BsuΔ*cysC* (Figure 5C). *Bacillus subtilis* switches its primary sulphur source between sulphate and methionine via compensatory regulation (Auger et al. 2002). This may account for the relatively high growth of BsuΔ*cysC* and Δ*mccAB* in the V8 medium or vegetable extracts, whereas BsuΔ*cysC*Δ*mccAB* exhibited significantly defective growth. Considering that BsuΔ*cysE* exhibited worse growth than BsuΔ*cysC*Δ*mccAB*, BsuΔ*cysC*Δ*mccAB* may synthesise cysteine from organic sulphur sources via sulphite in the V8 medium.

We then investigated the minimum amount of cysteine or methionine required for *B. subtilis* growth by adding cysteine or methionine to SP medium. BsuΔ*cysC* growth was most effectively restored, matching that of the WT, when 40 μM cysteine or 8 μM methionine was added (Figure 5D). Considering that the *E. coli* BW25113 Δ*cysC* mutant, in contrast, required 200 μM cysteine for maximum growth (Figure 3B), this indicates that *B. subtilis* has a metabolic pathway with high sulphur‐utilisation efficiency. Therefore, the ability of *B. subtilis* to acquire sulphur for cysteine synthesis from methionine, as well as from sulphate, along with its high sulphur‐utilisation efficiency, confers advantages for growth in plant extracts.

### 3.6 Sulphate‐Assimilation and Methionine‐to‐Cysteine Conversion Pathways Are Common in Plant‐Associated Bacteria

**FIGURE 6:**
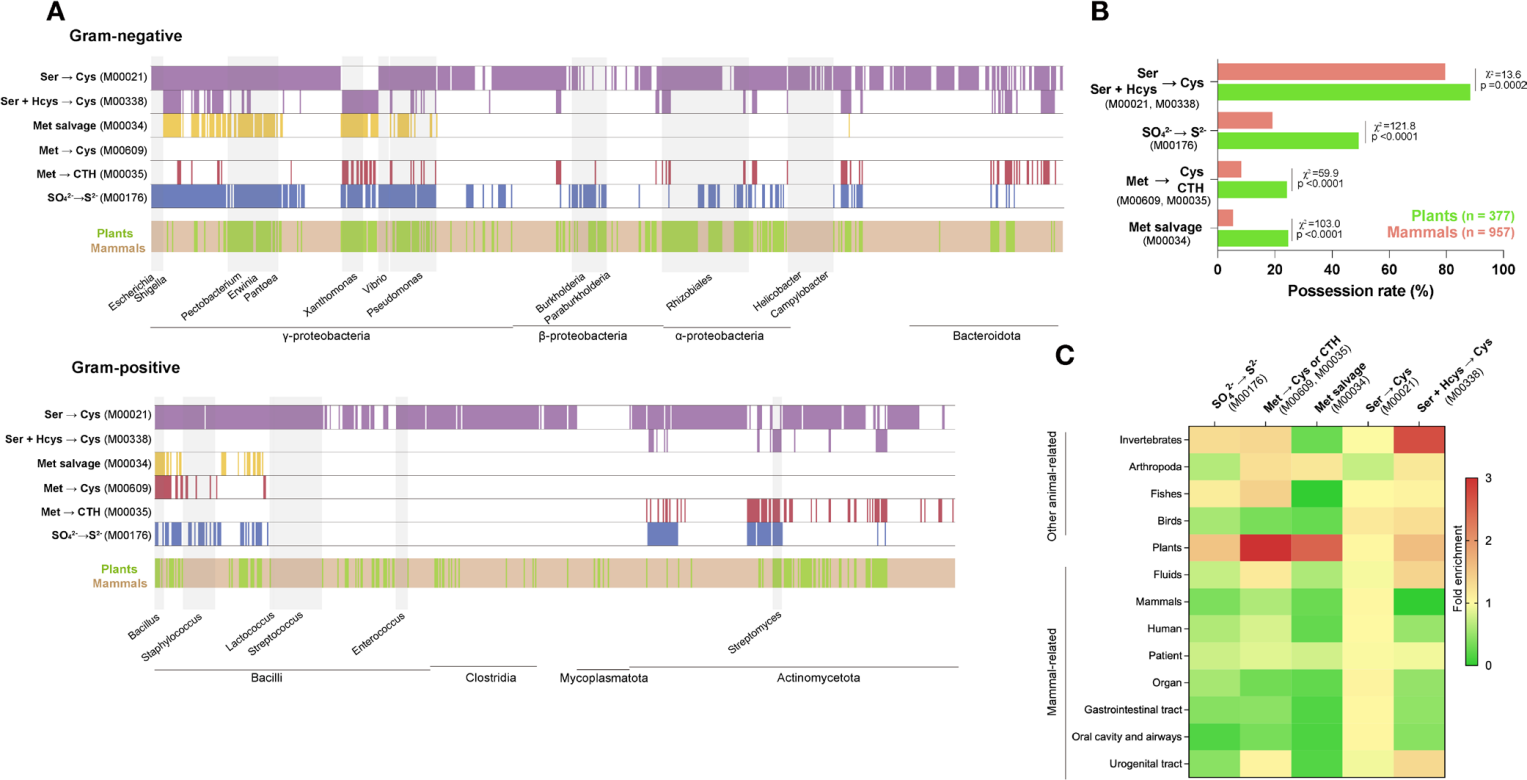
Pathways related to sulphur acquisition are more frequent in plant‐associated bacteria. (A) Distribution of pathways related to sulphur acquisition across bacteria. Sulphur assimilation (SO_4_^2−^ → S^2−^, KEGG module M00176, blue), methionine degradation (Met → CTH, M00035, red), cysteine synthesis from methionine (Met → Cys, M00609, red), methionine salvage (M00034, yellow), and cysteine biosynthesis (Ser → Cys, M00021; Ser + Hcys → Cys, M00338; purple) across plant‐associated bacteria (green) and mammal‐associated bacteria (brown). (B) Prevalence of each metabolic pathway in animal‐associated and plant‐associated bacteria. Green and brown bars indicate plant‐associated and animal‐associated bacteria, respectively. *χ* ^2^, Yates corrected Chi‐square. (C) Enrichment rates of each metabolic pathway across all isolate source categories. For the 3111 bacterial species annotated with habitat information, the proportion of bacteria possessing the target metabolic pathway within each habitat was divided by the proportion of bacteria in each habitat.

To clarify whether pathways related to sulphur acquisition confer a fitness advantage to bacteria for growth in plants, we investigated whether these pathways occur frequently in bacteria that primarily inhabit plants. Each bacterial species was examined for the presence of these metabolic pathways using the KEGG database, and major habitat information from the *BacDive* database was annotated. Of the 3111 bacterial species annotated with habitat information, 957 species were associated with mammals (occurring in body fluids, organs, or structures such the gastrointestinal tract and oral cavity) and 377 with plants (Table S3). This revealed that cysteine‐synthesis pathways, which use serine and sulphide or homocysteine as substrates (Ser → Cys and Ser + Hcys → Cys; KEGG modules M00021 and M00338, respectively), are conserved in a wide array of bacteria, with no major differences in their frequency between mammal‐ and plant‐associated bacteria (Figure 6A,B). The sulphate‐assimilation pathway (SO_4_^2−^ → S^2−^, M00176) occurs more commonly in plant‐associated than in mammal‐associated bacteria (Figure 6A,B). Although the pathways for cysteine synthesis from methionine (Met → Cys, M00609) occur exclusively in *Bacillus*, a few plant‐associated bacteria, including *Xanthomonas* and *Streptomyces*, possess both pathways for methionine degradation to cystathionine (Met → CTH, M00035) and cysteine synthesis via serine and homocysteine (Ser + Hcys → Cys, M00338) (Figure 6A). Bacterial species possessing these two metabolic pathways are likely to be able to convert methionine to cysteine. Among the other taxa studied here, the sulphate‐assimilation (M00176) and methionine‐degradation (M00609 or M00035) pathways are more common in the plant‐associated than in mammal‐associated bacteria. As an exception, these pathways were detected less frequently in species of *Rhizobiales*, which are intracellular parasites of plants. These bacteria may possess unique AA‐metabolic pathways, owing to their distinctive life cycles. The pathways for sulphate assimilation (M00176) and conversion of methionine to cysteine (M00609 or M00035) occur in 19.12% and 8.25%, respectively, of the mammal‐associated bacteria, and at significantly higher frequencies (49.33% and 24.13%, respectively) among the plant‐associated bacteria (Figure 6B). These findings highlight the importance of these pathways in acquiring and incorporating sulphur into cysteine for bacteria in plant‐derived environments.

We then analysed metabolic pathway enrichment based on bacterial habitat, revealing no significant differences among habitats in enrichment of the cysteine synthesis from serine and sulphide (M00021) pathway (Figure 6C). The enrichment ratios of the sulphate assimilation (M00176) and conversion of methionine to cysteine (M00609 or M00035) pathways were low (approximately 0.5) for most of the mammal‐ and bird‐associated habitats, but not for the fish‐, arthropod‐, or invertebrate‐associated habitats. In plants, the enrichment ratios of these pathways were high (at 2–3), suggesting that these metabolic pathways are strongly correlated with bacterial habitat in plants.

## 4 Discussion

In the present study, we demonstrated that sulphur acquisition for cysteine synthesis is a key requirement for bacterial growth in plant extracts. Cysteine plays critical roles in many cellular functions, including RNA modification, vitamin synthesis, protein redox sensing, and maintenance of protein tertiary structure (Giles et al. 2003; Barford 2004; Grazhdankin et al. 2020). In plants, intracellular free cysteine concentrations are maintained at low levels, because free cysteine is highly toxic; however, GSH synthesis during stress responses requires large quantities of free cysteine (Zagorchev et al. 2013; Pivato et al. 2014). To enable immediate synthesis of cysteine, plants efficiently uptake and utilise sulphate ions from the soil (Takahashi 2010, 2019), storing them in vacuoles (Kataoka et al. 2004). Given this ecological context, we predicted that the sulphate assimilation pathway is conserved in plant‐adapted bacteria, enabling them to grow better under the low‐cysteine conditions of plants. Although the sulphate assimilation pathway is essential in environments where sulphate ions are the main sulphur source, such as in natural waters (Zak et al. 2021), the sulphate assimilation pathway is also important in plant extracts, which are AA‐rich environments. Bacteria differ in their ability to utilise AAs; such differences have long been assumed to reflect the nutritional status of their major habitats. Various recent studies have examined the relationships between bacterial habitats (including plants) and AA requirements, primarily via large‐scale sequence and microbiome analyses (Ramoneda et al. 2023; Starke et al. 2023). However, little is known about the metabolic pathways that support bacterial growth in plant‐derived environments such as plant extracts and residues. Our findings thus provide important insights into bacterial nutrient acquisition in plant‐derived environments where systemic defence systems are not active, such as in plant residues and exudates.

*Escherichia coli* and *S. aureus* consume relatively large amounts of cysteine during their growth (Somerville and Proctor 2009; Yang et al. 2015). As *E. coli* is commonly found in the intestines, where AAs derived from digested or metabolised proteins are abundant (Dahm and Jones 1994; Circu and Aw 2012), it may possess metabolic pathways optimised for cysteine‐rich environments. Cysteine‐auxotrophic *E. coli* isolates are frequently isolated in clinical specimens (Borderon and Horodniceanu 1978; McIver and Tapsall 1993), suggesting that human organs are enriched in cysteine sources. As *E. coli* also occurs widely in other habitats, including soil and water (Figure 1C), the sulphate‐assimilation pathway may contribute to its growth in diverse environments with low cysteine levels. On the other hand, *S. aureus* typically colonises the skin and nasal cavity, where sweat and mucus are secreted; these body fluids, produced from blood plasma, contain considerable amounts of AAs, including cysteine and cystine (Gitlitz et al. 1974; Dunstan et al. 2016). Therefore, *S. aureus* may have evolved metabolic pathways that directly utilise cysteine or its derivatives. Consistent with this, *S. aureus* was detected less frequently than *E. coli* in non‐animal‐based environments (Figure 1C).

Various bacteria in the human body utilise GSH as a major cysteine source (Alkhuder et al. 2009; Lensmire et al. 2023). Although certain amount of GSH is present in plant cells, we found that the presence of large proportion of glutamine and glutamic acid inhibits the utilisation of GSH as a cysteine source by *E. coli* (Figure 3E). Studies using yeast have revealed that high concentrations of glutamate slow GSH degradation and facilitate its synthesis (Jaspers et al. 1985; Baudouin‐Cornu et al. 2012). The presence of glutamic acid (a constituent of GSH) and of its precursor, glutamine, may promote GSH synthesis in bacterial cells and inhibit GSH uptake and degradation. As sulphur sources for cysteine synthesis, various bacteria also utilise thiosulphate and taurine, which occur in the serum and other components of the human body (Lensmire and Hammer 2019), but are almost absent in plants (Figure S2) (Wang et al. 2008; Kawasaki et al. 2017). Sulphate and methionine may therefore be essential as sulphur sources for bacterial growth in plant extracts.

Methionine, like cysteine, is easily oxidised and relatively unstable. Methionine and cysteine are closely metabolically related, with cysteine providing the sulphur atom during methionine biosynthesis. As V8 medium exhibits low concentrations of methionine and cysteine (Figure 3A; Figure S2), we suspect that methionine deficiency may hinder bacterial growth in plant extracts. We found that the methionine‐salvage pathway was detected more frequently in plant‐associated bacteria, including *B. subtilis* , *P. carotovorum* , and *X. campestris* , than in animal‐associated bacteria (Figure 6). The methionine‐salvage pathway, which produces methionine via the efficient recycling and utilisation of sulphur‐containing metabolites generated by other metabolic pathways (Sekowska et al. 2004; Albers 2009), may assist bacteria in growing efficiently in plant extracts, which are conditions of limited availability of sulphur‐containing AAs.

*Escherichia coli* O157:H7 Sakai grew significantly better in V8 medium than *E. coli* BW25113 (Figure 1D), implying that it has a lower cysteine requirement and is thus better adapted to plants as a habitat. *Escherichia coli* O157:H7 Sakai has caused outbreaks by contaminating radish sprouts, and *E. coli* O157 outbreaks typically result from contaminated vegetables (Michino et al. 1999; Tabuchi et al. 2015; Sai and Balachandhar 2019). The low cysteine requirements of this strain may enable it to survive and grow on plants and vegetables. Compared to the genome of *E. coli* K‐12, that of *E. coli* O157 contains an additional 1.4 Mb exogenous gene region (Hayashi 2001). We speculate that this region contains genes that reduce its cysteine requirements and enhance its sulphur‐utilisation efficiency.

To simplify our experimental design and improve throughput, we used a medium based on V8 juice. The V8 medium and vegetable extracts exhibited similar AA composition (Figures S2B and S5), and the importance of sulphur acquisition in cysteine synthesis was revealed for many of the vegetable extracts (Figure 4). However, V8 juice contains additional vegetable‐derived compounds such as citric acid, ascorbic acid, and beta‐carotene, potentially affecting our screening and GO enrichment results.

Furthermore, we performed genetic screening using *E. coli* , which is not one of the most representative bacteria in plant microbiomes. However, this approach offers two advantages: First, genetic tools and information are readily available for *E. coli* , as it is one of the most well‐studied bacteria; and second, as *E. coli* is not fully adapted to plants, its use enables us to elucidate the fundamental mechanisms to grow in plant‐derived environments. Using *E. coli* thus revealed that sulphate assimilation for cysteine acquisition is important for bacterial adaptation to plants. This outcome would not have been obtained if we had screened using bacterial species, such as *B. subtilis* or plant‐pathogenic bacteria, that can obtain sulphur for cysteine biosynthesis from multiple sulphur sources. Nonetheless, gene screening using additional plant‐adapted bacterial species should be performed in the future, as it may reveal specialised nutrient acquisition systems for plants.

## 5 Conclusions

Our findings, based on gene screening using *E. coli* cultured in V8 medium, reveal that the sulphate‐acquisition pathway is important for bacterial growth in plant extracts, owing to their low levels of free cysteine. Although plants contain high concentrations of the cysteine‐containing peptide glutathione, we found that the acquisition of cysteine from glutathione is inhibited in the presence of polar AAs. Further, we found that bacteria living in plant‐based habitats frequently possess pathways for cysteine synthesis from either sulphate or methionine. These findings suggest that the sulphur‐acquisition pathway for cysteine synthesis contributes fundamentally to bacterial fitness in plant‐based environments.

## Author Contributions

K.I. and C.K. conceptualised the study. K.I. planned experiments. K.I., S.Y., T.T. and M.T. performed experiments. K.I. and M.T. performed data analysis. K.I. wrote the first draft of the manuscript. S.Y., K.F. and C.K. supervised the experiments and writing. All authors discussed the results and the manuscript.

## Ethics Statement

The authors have nothing to report.

## Conflicts of Interest

The authors declare no conflicts of interest.

## Supporting information


**Figure S1.** Genotyping and confirmation of the cysteine‐auxotrophic phenotype of the mutants used in this study. (A) Genotyping of *Escherichia coli* cysteine biosynthesis‐deficient mutants by PCR. (B) Confirmation of cysteine requirement of Δ*cysB* (JW1267‐KC). (C) Generation of Δ*cysB* by one‐step inactivation method. (D) Genotyping of BsuΔ*cysC*, BsuΔ*cysE*, and BsuΔ*cysC*Δ*mccAB* by PCR.

**Figure S2.** Total protein, amino acid, and sugar contents in lysogeny broth (LB), V8 medium, and foetal bovine serum (FBS). (A) Total protein contents. Data are shown as means ± SD. (B) Free amino acid contents was analysed by HPLC using pre‐column labelling with 4‐fluoro7‐nitro‐2,1,3‐benzoxadizol (NBD‐F). (C) Summary of the composition of LB, V8 medium, and FBS. Sugar content was evaluated according to the kit manufacturer’s instructions and published methods (Sezonov et al. 2007; Kent et al. 2008).

**Figure S3.** Cysteine auxotrophic phenotype and growth, in lysogeny broth (LB) and V8 medium, of mutants defective in cysteine biosynthesis. (A) *E. coli* BW25113 (Parent) and cysteine auxotrophic mutants were spotted onto M9 agar with or without 0.02% (w/v) l‐cysteine. Bacteria were incubated at 37°C for 16 h (for Δ*cysA*, Δ*cysC*, Δ*cysG*, Δ*cysH*, Δ*cysK*, Δ*cysP*, Δ*cysQ*, Δ*cysT*, and Δ*cysW*) or 24 h (for Δ*cysB* and Δ*cysE*). (B) Growth of BW25113 (Parent) and cysteine‐auxotrophic mutants in LB or V8 medium at 30°C. Coloured lines and areas show the mean ± standard deviations (SD), *n* = 3.

**Figure S4.** Growth of Δ*cysC* in M9 medium with cystine. Cystine was added to M9 medium at the indicated concentrations and growth was examined at 30°C. Date are shown as means ± SD, *n* = 3.

**Figure S5.** Amino acid quantification in vegetables via HPLC using pre‐column labelling with amine‐reactive 4‐fluoro‐7‐nitro‐2,1,3‐benzoxadizole (NBD‐F).

**Figure S6.** Cysteine‐auxotrophic phenotype of *Bacillus subtilis* mutants defective in cysteine biosynthesis on Spizizen minimal medium agar (SP).

**Table S1.** Oligonucleotides used in this study.

**Table S2.** Output of Gene Ontology (GO) enrichment analysis of deleted genes that negatively affected bacterial growth in V8 medium.

**Table S3.** List of bacterial isolation sources and distribution of each metabolic pathway.

## Data Availability

The raw data were generated at Okayama University. The derived data supporting the findings of this study are available from the corresponding author C.K. on request.
