# Supplementary figures and images for: Sulphur‐Acquisition Pathways for Cysteine Synthesis Confer a Fitness Advantage to Bacteria in Plant Extracts
Source: Environ Microbiol. 2025 Jun 17;27(6):e70126. doi: 10.1111/1462-2920.70126 (PMC12174637; doi:10.1111/1462-2920.70126)

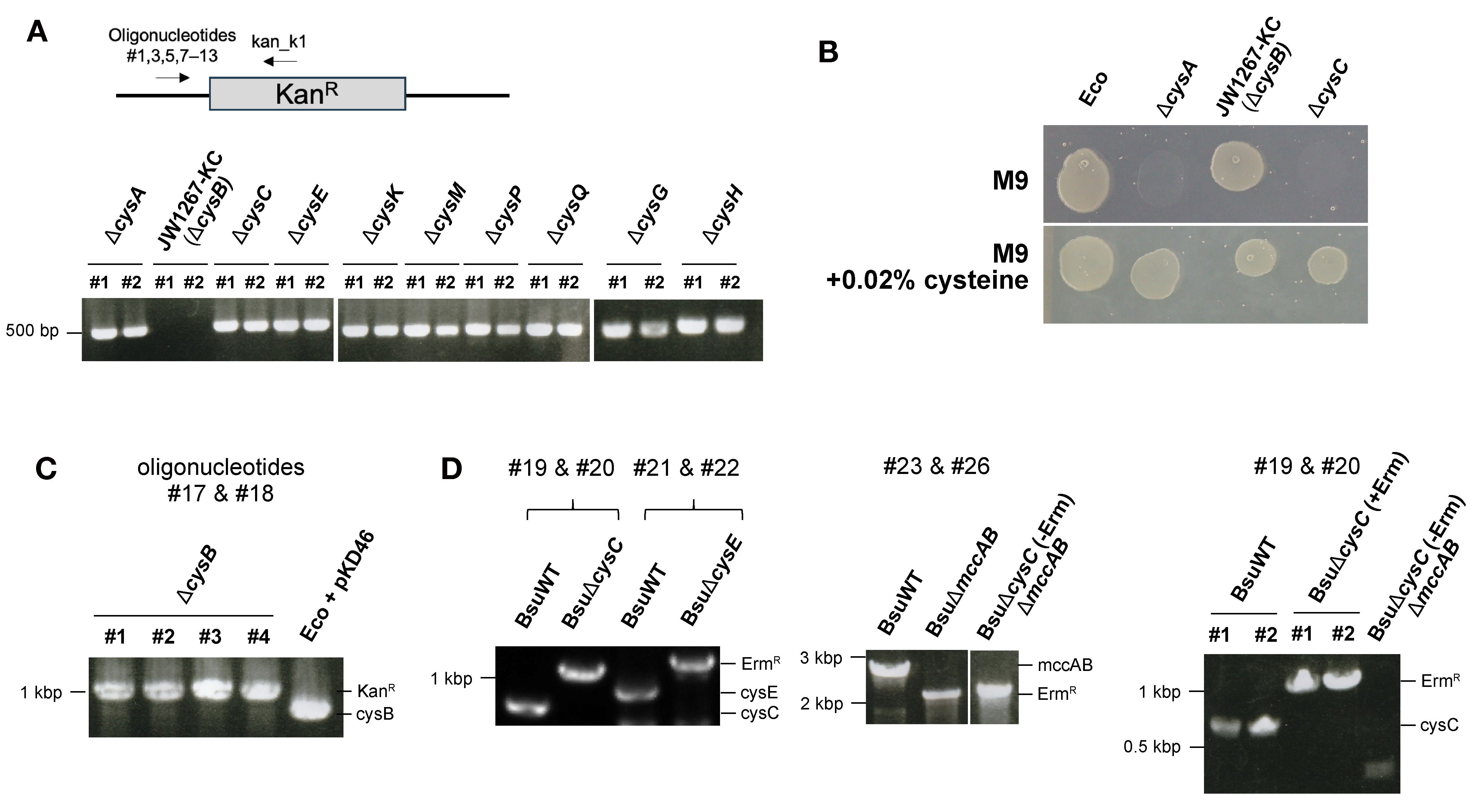

Supplement: Supplementary file 1 — Figure S1. Genotyping and confirmation of the cysteine‐auxotrophic phenotype of the mutants used in this study. (A) Genotyping of Escherichia coli cysteine biosynthesis‐deficient mutants by PCR. (B) Confirmation of cysteine requirement of ΔcysB (JW1267‐KC). (C) Generation of ΔcysB by one‐step inactivation method. (D) Genotyping of BsuΔcysC, BsuΔcysE, and BsuΔcysCΔmccAB by PCR. [file EMI-27-e70126-s001.tif]

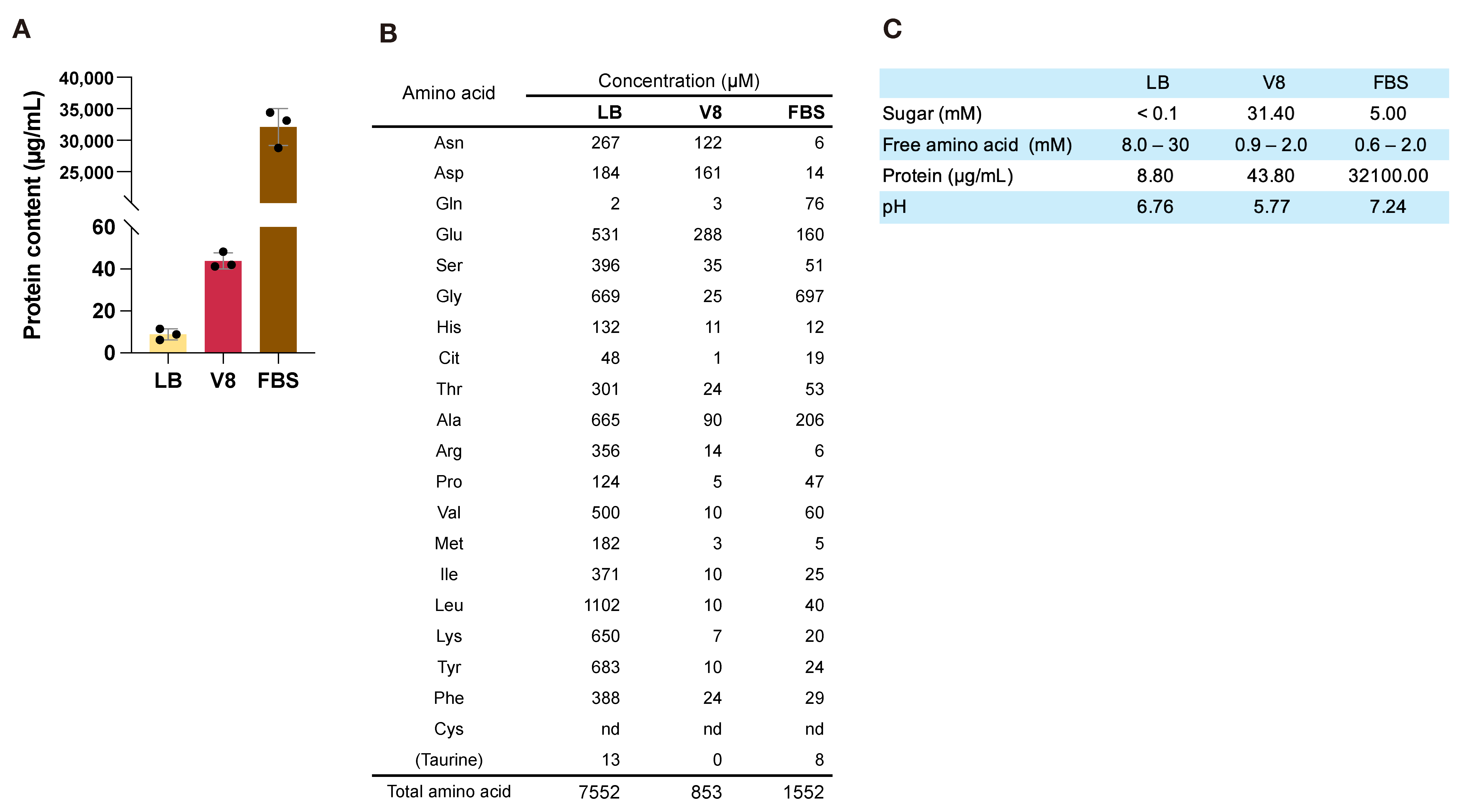

Supplement: Supplementary file 2 — Figure S2. Total protein, amino acid, and sugar contents in lysogeny broth (LB), V8 medium, and foetal bovine serum (FBS). (A) Total protein contents. Data are shown as means ± SD. (B) Free amino acid contents was analysed by HPLC using pre‐column labelling with 4‐fluoro7‐nitro‐2,1,3‐benzoxadizol (NBD‐F). (C) Summary of the composition of LB, V8 medium, and FBS. Sugar content was evaluated according to the kit manufacturer’s instructions and published methods (Sezonov et al. 2007; Kent et al. 2008). [file EMI-27-e70126-s009.tif]

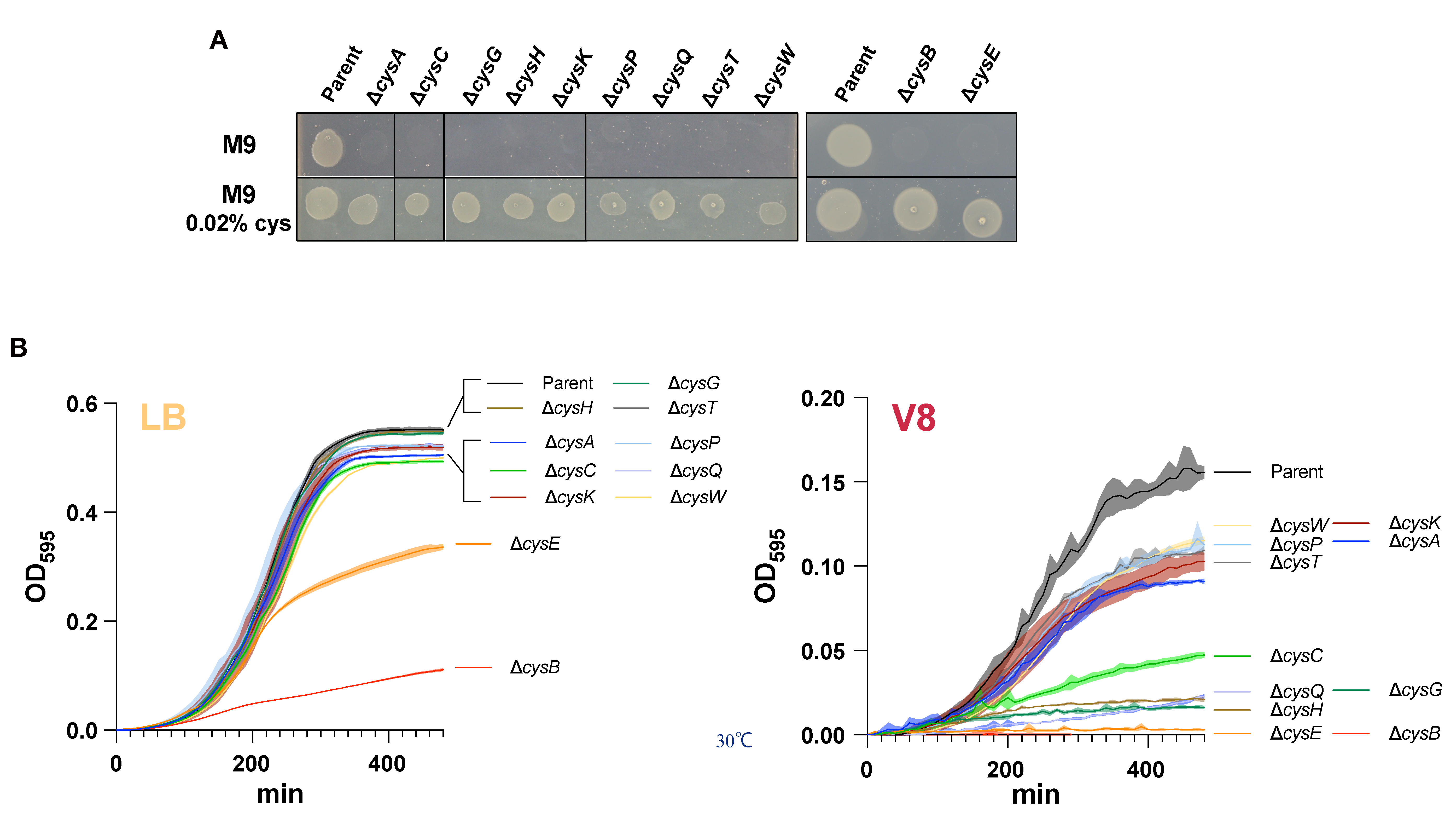

Supplement: Supplementary file 3 — Figure S3. Cysteine auxotrophic phenotype and growth, in lysogeny broth (LB) and V8 medium, of mutants defective in cysteine biosynthesis. (A) E. coli BW25113 (Parent) and cysteine auxotrophic mutants were spotted onto M9 agar with or without 0.02% (w/v) l‐cysteine. Bacteria were incubated at 37°C for 16 h (for ΔcysA, ΔcysC, ΔcysG, ΔcysH, ΔcysK, ΔcysP, ΔcysQ, ΔcysT, and ΔcysW) or 24 h (for ΔcysB and ΔcysE). (B) Growth of BW25113 (Parent) and cysteine‐auxotrophic mutants in LB or V8 medium at 30°C. Coloured lines and areas show the mean ± standard deviations (SD), n = 3. [file EMI-27-e70126-s004.tif]

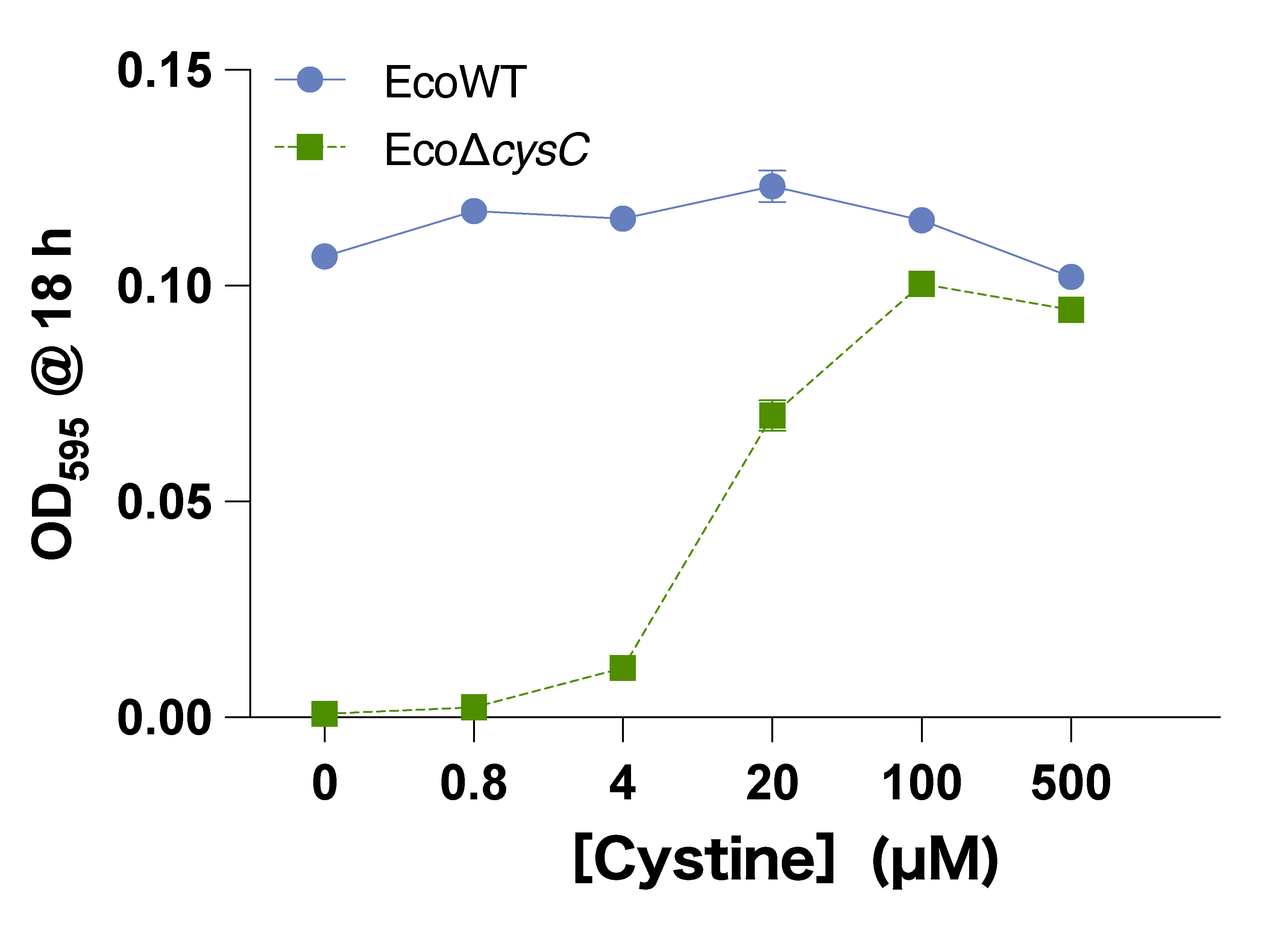

Supplement: Supplementary file 4 — Figure S4. Growth of ΔcysC in M9 medium with cystine. Cystine was added to M9 medium at the indicated concentrations and growth was examined at 30°C. Date are shown as means ± SD, n = 3. [file EMI-27-e70126-s003.tif]

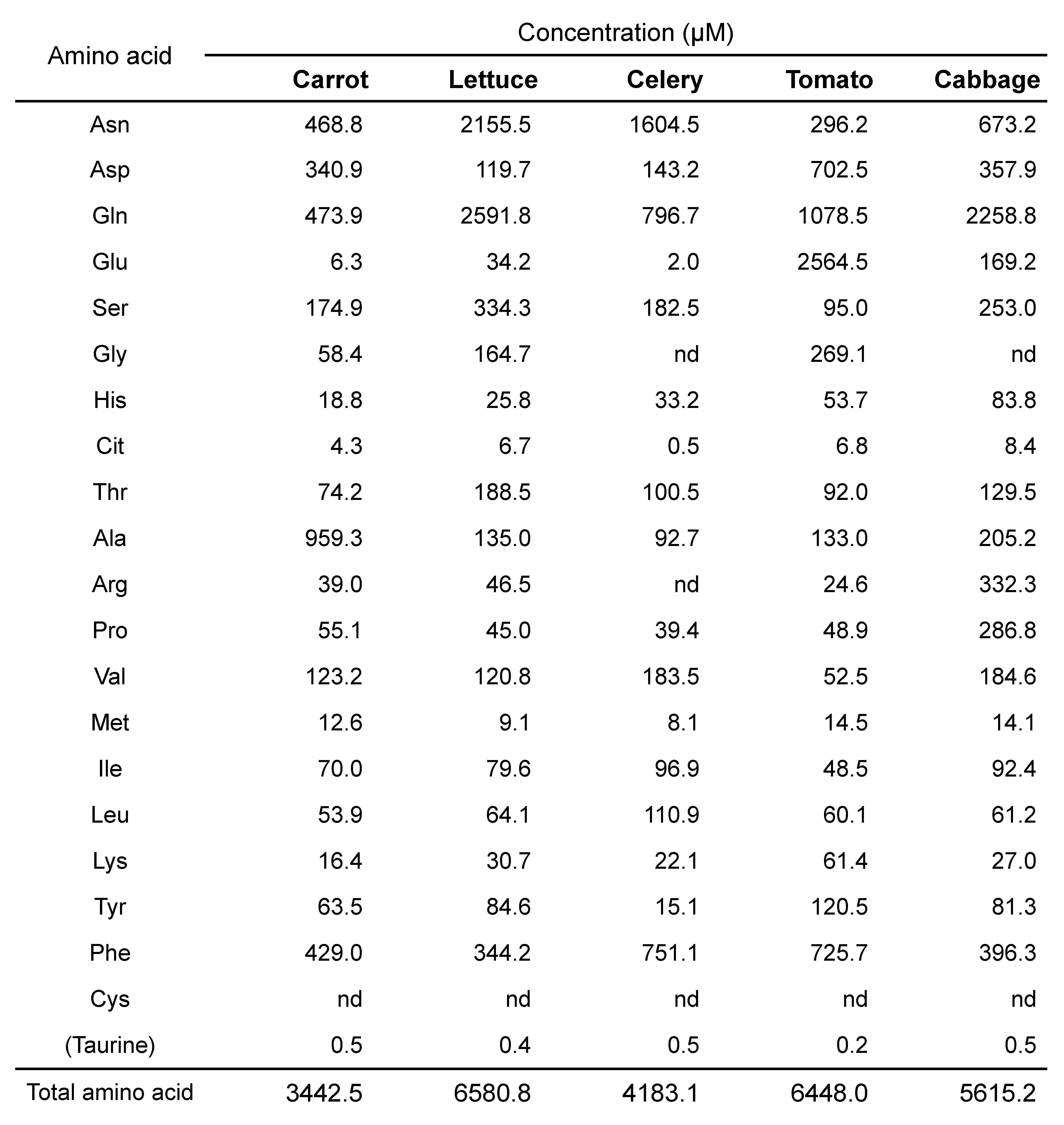

Supplement: Supplementary file 5 — Figure S5. Amino acid quantification in vegetables via HPLC using pre‐column labelling with amine‐reactive 4‐fluoro‐7‐nitro‐2,1,3‐benzoxadizole (NBD‐F). [file EMI-27-e70126-s005.tif]

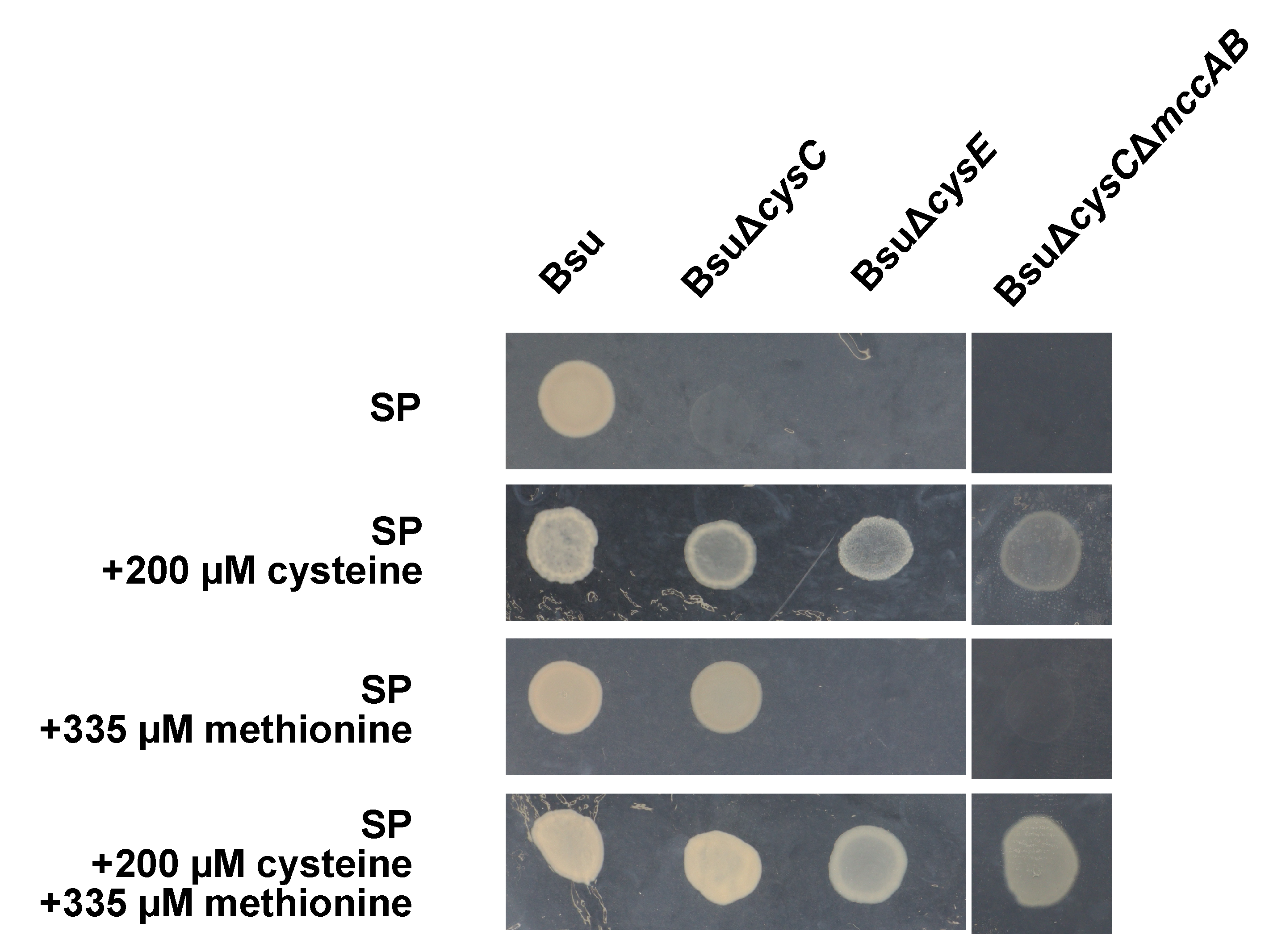

Supplement: Supplementary file 6 — Figure S6. Cysteine‐auxotrophic phenotype of Bacillus subtilis mutants defective in cysteine biosynthesis on Spizizen minimal medium agar (SP). [file EMI-27-e70126-s007.tif]
